# The Relationship Between Suicidal Ideation and Parental Attachment Among Adolescents: The Mediator of Anhedonia and Peer Attachment

**DOI:** 10.3389/fpsyg.2021.727088

**Published:** 2021-10-18

**Authors:** Yaru Guo, Yifu Ji, Yunheng Huang, Man Jin, Yanting Lin, Yun Chen, Lei Zhang, Chunyan Zhu, Fengqiong Yu, Kai Wang

**Affiliations:** ^1^Institute of Mental Health and Psychological Science, Anhui Medical University, Hefei, China; ^2^Psychiatry Department of Hefei Fourth People's Hospital, Hefei, China; ^3^Anhui Xinyu Psychological Service, Hefei, China; ^4^Department of Mental Health and Psychological Science, Anhui Province Key Laboratory of Cognition and Neuropsychiatric Disorders, Anhui Medical University, Hefei, China

**Keywords:** parental attachment, peer attachment, anhedonia, suicidal ideation, structural equation model

## Abstract

**Background:** Previous studies had shown that poor quality of early parental attachment is a risk factor for suicide, but few have focus on the mechanism between suicidal ideation and parental attachment. The aim of this study was to explore how parental attachment, anhedonia, and peer attachment were associated with suicidal ideation in adolescents.

**Method:** Participants were enrolled in middle schools, in Hefei, Anhui, China. All participants completed socio-demographic characteristic and standard assessments on parental attachment, peer attachment, anhedonia, and suicidal ideation by paper surveys. The effect of parental attachment on suicidal ideation mediated by anhedonia and peer attachment was analyzed by a structural equation model (SEM) using SPSS AMOS 23.0.

**Results:** The SEM analysis revealed that the standard total effect of parental attachment on suicidal ideation was −0.137 (*Z*=−27.00, 95% confidence interval [CI; −0.147, −0.127], *p*<0.001), with a direct effect of parental attachment on suicidal ideation of −0.107 (*Z*=−21.40, 95% CI [−0.117, −0.098], *p*<0.001), while the indirect effects were−0.002 (*Z*=−3.33, 95% CI [−0.003, −0.002], *p*<0.001) in the pathway of parental attachment-anhedonia-peer attachment-suicidal ideation, −0.019 (*Z*=−19.00, 95%CI [−0.022, −0.017], *p*<0.001) in the pathway of parental attachment-anhedonia-suicidal ideation, and−0.008 (*Z*=−7.00, 95% CI [−0.010, −0.007], *p*<0.001) in the pathway of parental attachment-peer attachment-suicidal ideation.

**Conclusion:** The study suggested that parental attachment could directly influence suicidal ideation and indirectly influence suicidal ideation via anhedonia and peer attachment. The results emphasized the importance of attachment in infancy and verified the feasibility of intervention on anhedonia and peer attachment to prevent suicidal ideation.

## Introduction

For teenagers, suicide is a major public health concern and remains a leading cause of mortality worldwide (Kutcher and Szumilas, 2008). Studies suggested that suicidal ideation can better predict completed suicide (Posner et al., 2011). As a major suicidal risk factor, suicidal ideation is common with prevalence rates ranging from 6 to 25.0% among United States adolescents and 2.7 to 45.1% among Chinese adolescents (Lew et al., 2020). Although the higher suicidal ideation prevalence rates for Chinese adolescents, they showed less for help-seeking (Liu et al., 2018). In addition, consistent with previous studies, Lew’s research indicated that both United States and Chinese female adolescents showed relatively higher suicidal ideation as compared to male (Grunbaum et al., 2004; Zhang et al., 2011; Lew et al., 2020). For the discrepancy of suicidal ideation, previous studies suggested that it could be attributed to complex influences of personal, social, environmental, and developmental factors (Hassan, 1998; Ernst et al., 2009; Van Orden et al., 2010). According to Adam’s development model, early adverse parenting experiences could lead to insecure parental attachment that could then become a risk factor of suicidal ideation and negative behaviors later in life (Adam et al., 1996). Especially in China, the attached great importance to family cohesion highlights the role of attachment for suicidal ideation (Lew et al., 2020). In addition, due to Confucian ethics, Chinese adolescents have been carefully groomed and molded to subordinate to certain societal norms and are obliged to fulfil the expectations of their elders, family, and society (Jia and Zhang, 2017; Lew et al., 2020). This added pressure of social interactions exacerbates the occurrence of suicidal ideation. Thus, in this study, we considered the onset of suicidal ideation from the perspective of attachment.

Attachment is a deep and enduring emotional bond that is established in a variety of different relationships, such as parental attachment, peer attachment, and romantic partner attachment (Ainsworth, 1979; Bergman et al., 2013). Attachment theory suggests that each type of attachment may be influenced by the primary type of attachment, parental attachment (Bartholomew and Horowitz, 1991; Bergman et al., 2013). Specifically, experiences with parents during infancy directly influence perceived emotional valences/support, protection, and security (Mikulincer et al., 2013) and indirectly construct the working model of self and others (Bartholomew and Horowitz, 1991). Studies have concluded that individuals with the absence of secure parental attachment fail to learn self-regulatory strategies, which lead to negative and destructive behavior toward their peer partners (Taylor, 2010; Cerutti et al., 2018). Furthermore, studies verified that individuals with poor quality of attachment were reluctant to seek out relationships with others (Bartholomew and Horowitz, 1991; Mikulincer et al., 2013) and that these unmet interpersonal relationships may contribute to eventual suicidal ideation (Nagra et al., 2016; Bar-Zomer and Brunstein Klomek, 2018). Taken together, the data from the above studies showed that both parental and peer attachment may impact suicidal ideation. However, adolescents are influenced more by peer attachment during this time period as interactions with peer partners increase at the expense of parental attachment (Thorlindsson and Bernburg, 2009); therefore, prevention of suicidal ideation can be better applied from a peer attachment rather than parental attachment standpoint. Here, we aimed to explore the direct influence of parental attachment and indirect influence of peer attachment on suicidal ideation.

Studies had already reached a consensus that anhedonia is a major risk factor of suicidal ideation (Hawes et al., 2018; Loas et al., 2018). Individuals with anhedonia showed greater possibility of suicidal ideation (Zielinski et al., 2017). Anhedonia is characterized as the loss of pleasure or lack of reactivity to pleasurable stimuli and includes anticipatory anhedonia and consummatory anhedonia, both of which show stable individual differences (Herbener et al., 2005). Neurobiological studies have identified a common association between anhedonia and suicidal ideation. Anticipatory anhedonia and suicidal ideation were found to be linked to a decreased dopamine pathway and striatal circuitry (Berridge and Kringelbach, 2008; Vang et al., 2010), and consummatory anhedonia and suicidal ideation were linked to serotonin and opioid system dysfunction in the amygdala and the ventromedial prefrontal cortex (Kennedy et al., 2006; Meerwijk et al., 2013). Furthermore, Yang et al. found that anhedonia was a strong mediator that could indirectly influence suicidal ideation (Yang et al., 2020). Several studies verified that individuals with anhedonia showed increased social isolation and experienced less emotional expressiveness, worse social adjustment, and increased avoidance of reality (Joiner et al., 2005; Lussier and Loas, 2015). This long-term social isolation in adolescents may contribute to insecure peer attachment that results in eventual suicidal ideation (Winer et al., 2014). The enduring, stable characteristics of anhedonia (Chapman et al., 1994; Kwapil et al., 1997; Herbener and Harrow, 2002) led us to hypothesize that it could increase the likelihood of eventual suicidal ideation.

**Figure 1:**
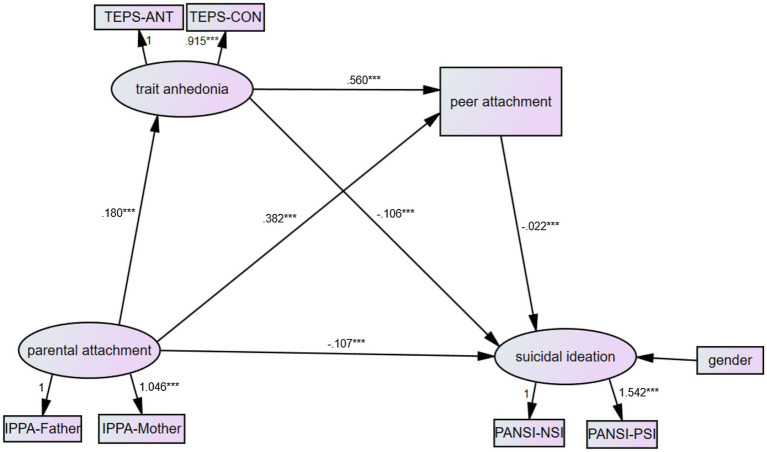
Final model with the standardized coefficients and unstandardized coefficients presented in the parentheses. ^*^p<0.05, ^**^p<0.01, ^***^p<0.001.

Despite many studies on suicide, those exploring the complex relationships among suicidal ideation, parental attachment, peer attachment, and anhedonia are non-existent, and based on this, we set up a hypothetical model using a structural equation model (SEM). In this hypothetical model, we hypothesized that parental attachment would directly influence suicidal ideation and indirectly influence suicidal ideation via anhedonia and peer attachment (Figure 1).

## Materials and Methods

### Participants

In total, 10,400 students were enrolled in the current study from the Feidong 1 middle school, Feidong 3 middle school, Feidong 10 middle school, and Lujiang middle school, Hefei, China, between December 2019 and January 2020. Out of the 10,400 students, 8,680 completed the questionnaires by paper surveys. All eligible students signed the informed consents. Ethical approval was obtained from Anhui medical university ethics committee (NCT03991572).

### Materials

Socio-demographic characteristics of adolescents were collected, including gender, age, grade, family life, amount of siblings, and clinically diagnosed mental illness history (themselves and their family), as well as the history of non-suicidal self-injury (NSSI).

### Positive and Negative Suicide Ideation Inventory

The positive and negative suicide ideation (PANSI) inventory consists of 14 items on a 5-point Likert scale, which are scored from 1 (never) to 5 (most of the time). It covers two types of suicide ideation during a 2-week period: negative suicide ideation (PANSI-NSI), which has eight items, and positive suicide ideation (PANSI-PSI), which consists of six items. The sum of the PANSI score ranged from 14 to 70 points (PANSI>42 were referred as suicidal ideation, and PANSI≤42 were defined as non-suicidal ideation), with a higher score suggestive of a higher severity of suicidal ideation (Osman et al., 1998). The Chinese version of the PANSI was widely used in the current study. It showed good psychometric properties, and Cronbach’s alpha coefficients for the PANSI-PSI and PANSI-NSI were 0.94 and 0.86, respectively (Chang et al., 2009).

### Inventory of Parent and Peer Attachment

The inventory of parent and peer attachment (IPPA) is used to measure the intimate relationship between adolescents and their parents and peers. It covers three types of attachments: perceived quality of father-adolescent attachment (IPPA-Father), perceived quality of mother-adolescent attachment (IPPA-Mother), and the perceived quality of friend-adolescent attachment (IPPA-Friend). Each of the three subscales has 25 items, on a 5-point Likert scale ranging from 1 (almost never true) to 5 (almost always true), with a higher total score indicative of a better perceived quality of attachment (Armsden and Greenberg, 1987). The IPPA is widely used for adolescents aged from 12 to 19years, and Cronbach’s alphas of the Chinese version ranged from 0.75 to 0.88 for father-adolescent attachment, from 0.76 to 0.91 for mother-adolescent attachment, and from 0.64 to 0.90 for peer attachment (Zou et al., 2020).

### Temporal Experience of Pleasure Scale

The level of trait anhedonia was measured by the temporal experience of pleasure scale (TEPS). The inventory consists of 20 items on a 6-point Likert scale, which are scored from 1 (totally false for me) to 6 (totally true for me). It includes two subscales: trait anticipatory anhedonia (Trait-Ant) and trait consummatory anhedonia (Trait-Con). The total score of the TEPS ranges from 20 to 120, with a lower score indicative of a more severe level of trait anhedonia (Gard et al., 2007; Chan et al., 2010). The Chinese version of the TEPS has recently been verified with Cronbach’s alpha for Trait-ANT and Trait-CON of 0.84 and 0.85, respectively (Yang et al., 2020).

### Statistical Analysis

The data were analyzed using SPSS and AMOS version 21.0. We first ran the descriptive statistics of the socio-demographic characteristics in adolescents. Then, Pearson’s correlation tests were performed to explore the associations among IPPA-Father, IPPA-Mother, IPPA-Friend, TEPS-Ant, Trait-Con, PANSI-PSI, and PANSI-NSI. Finally, we conducted the SEM using AMOS 21.0. The significance level was set as *α*=0.05 (two-tailed) in all data analyzed.

## Results

### Sample Characteristics

The 8,680 adolescents aged from 12 to 19years old (Mean=15.224, *SD*=2.559). Concrete socio-demographic characteristics of them are shown in Table 1. The male-to-female ratio was approximately 50:50. About three-fourths of the adolescents were live in urbans areas and had siblings. Overall, 11.1% of adolescents reported a clinical diagnosis of mental illness, and 9.4% reported a confirmed family history of mental illness. Among all of the adolescents, 1993 were junior (age: mean=13.56, *SD*=1.510) and 6,687 were senior (age: Mean=15.74, *SD*=2.595). *t* Test showed no significant difference of junior/senior on suicidal ideation [*t*(8678)=1.533, *p*>0.1]. Finally, detection rates of 48.8 and 4.9% were reported for NSSI and suicidal ideation, respectively.

**Table 1:** Socio-demographic characteristics of adolescents.

Characteristic	number	Percent (%)
Gender		
Men	4626	53.2
Women	4054	46.8
Grade		
Seven	908	10.4
Eight	1085	12.5
Ten	3430	39.5
Eleven	3257	37.6
Family live		
City	6490	74.7
Rural	2190	25.3
Being the only child		
Yes	2681	30.9
No	5999	69.1
History of mental illness		
Yes	960	11.1
No	7720	88.9
History of family mental illness		
Yes	811	9.4
No	7869	90.6
Non-suicidal self-injury		
Yes	4240	48.8
No	4440	51.2
Suicidal ideation		
Yes	417	4.9
No	8263	95.1

### Descriptive Statistics

Results from the correlation analysis are shown in Table 2. Suicidal ideation was significantly correlated with anhedonia [(*r*_PANSI-PSI*TEPS-ANT_=−0.400, *r*_PANSI-PSI*TEPS-CON_=−0.344, *r*_PANSI-NSI*TEPS-CON_=−0.187, *r*_PANSI-NSI*TEPS-CON_=−0.124), *p*<0.001] and attachment [(*r*_PANSI-PSI*IPPA-Father_=−0.439, *r*_PANSI-PSI*IPPA-Mother_= −0.485, *r*_PANSI-PSI*IPPA-Friend_=−0.399, *r*_PANSI-NSI*IPPA-Father_=−0.322, *r*_PANSI-NSI*IPPA-Mother_=−0.352, *r*_PANSI-NSI*IPPA-Friend_=−0.244), *p*<0.001]. Anhedonia was also significantly correlated with attachment [(*r*_TEPS-ANT*IPPA-Father_=0.226, *r*_TEPS-ANT*IPPA-Mother_=0.257, *r*_TEPS-ANT*IPPA-Friend_=0.289, *r*_TEPS-CON*IPPA-Father_=0.194, *r*_TEPS-CON*IPPA-Mother_=0.232, *r*_TEPS-CON*IPPA-Friend_=0.244), *p*<0.001].

**Table 2:** Correlations among subscales of the inventory of parent and peer attachment (IPPA), temporal experience of pleasure scale (TEPS), and Positive and negative suicide ideation (PANSI).

	PANSI-PSI	PANSI-NSI	IPPA-Father	IPPA-Mother	IPPA-Friend	TEPS-ANT	TEPS-CON
PANSI-PSI	1						
PANSI-NSI	0.477^***^	1					
IPPA-Father	−0.439^***^	−0.322^***^	1				
IPPA-Mother	−0.485^***^	−0.352^***^	0.567^***^	1			
IPPA-Friend	−0.399^***^	−0.244^***^	0.315^***^	0.321^***^	1		
TEPS-ANT	−0.400^***^	−0.187^***^	0.226^***^	0.257^***^	0.289^***^	1	
TEPS-CON	−0.334^***^	−0.124^***^	0.194^***^	0.232^***^	0.244^***^	0.597^***^	1

*** *p*<0.001;

^**^p<0.01; ^*^p<0.05.

For previous studies showed the gender difference, an additional *t* test was conducted to explore the gender difference in suicidal ideation with results indicating that females [PANSI-PSI (*M*=15.98, *SD*=4.58); PANSI-NSI (*M*=11.44, *SD*=5.05)] relative to males [PANSI-PSI (*M*=14.92, *SD*=4.57); PANSI-NSI (*M*=10.39, *SD*=4.17)] showed significantly higher suicidal ideation [PANSI-PSI, *t*(8678)=−10.828, *p*<0.001]; [PANSI-NSI, *t*(8678)=−10.655, *p*<0.001]. The significant gender difference in suicidal ideation indicated the need to control for it while conducting the subsequent SEM.

### Mediation Analysis

An overall SEM of the theoretical relationships is shown in Figure 1. The study results indicated an adequate model fit [*χ*^2^(15)=34.94, *p*<0.001, CFI=0.970, RMSEA=0.063; Figure 1]. The SEM analysis revealed that the standard total effect of parental attachment on suicidal ideation was −0.137 (*Z*=−27.00, 95% CI [−0.147, −0.127], *p*<0.001), with a direct effect of parental attachment on suicidal ideation of −0.107 (*Z*=−21.40, 95% CI [−0.117, −0.098], *p*<0.001); indirect effects of −0.002 (*Z*=−3.33, 95% CI [−0.003, −0.002], *p* <0.001) in the pathway of parental attachment-trait anhedonia-peer attachment-suicidal ideation; −0.019 (*Z*=−19.00, 95%CI [−0.022, −0.017], *p*<0.001) in the pathway of parental attachment-trait anhedonia-suicidal ideation; and −0.008 (*Z*=−7.00, 95% CI [−0.010, −0.007], *p*<0.001) in the pathway of parental attachment-peer attachment-suicidal ideation. In addition, the two mediators, anhedonia and peer attachment, were significantly related (*r*=0.560, *p*<0.001).

## Discussion

The purpose of this study was to explore how parental attachment, anhedonia, and peer attachment predict suicidal ideation. Based on the SEM, our results suggested that parental attachment could influence suicidal ideation directly and indirectly *via* anhedonia and peer attachment. Thus, anhedonia and peer attachment can partially mediate the effect of parental attachment on suicidal ideation. The mediation framework was meaningful and instructional for prevention of suicidal ideation by insights about attachment and anhedonia.

In the current study, the prevalence of suicidal ideation was 4.9%. And since the measured suicidal ideation with a period of at least 2weeks, which highlighted the immediate focus and/or intervention for individuals with high suicidal ideation scores. Consistent with previous studies, our results also showed a gender difference in suicidal ideation (Grunbaum et al., 2004; Zhang et al., 2011) and suggest that intervention policies may need to be gender-specific. Worth noting, we did not find the difference of suicidal ideation on junior and senior students, which was converse to one previous study, it showed a sharp increase in suicidal ideation in senior aged from 15–18 years for western students (Berger et al., 2015). But here, we guess that it may contribute to the incomplete sample for the not measured entrance students in our research.

Our results suggested that parental attachment can directly influence suicidal ideation and indirectly influence suicidal ideation via peer attachment. Consistent with previous studies, individuals with lower quality of parental attachment are more likely to have suicidal ideation (Cerutti et al., 2018; Gandhi et al., 2019). However, the significant effect of peer attachment on suicidal ideation in the pathway of parental attachment-peer attachment-suicidal ideation in our results is novel. We also found that the indirect effect on suicidal ideation in this pathway was consistent with attachment theory, in which it is suggested that peer attachment is influenced by parental attachment (Bartholomew and Horowitz, 1991; Bergman et al., 2013). Attachment theory proposes that individuals with insecure parental attachment are more likely to construct a poor quality of peer attachment. Furthermore, the interpersonal theory of suicide states that the thought of suicide may originate in the unmet interpersonal needs as indexed by peer attachment (Loas et al., 2018). Thus, it may be that an insecure attachment of adolescents with their parents can negatively influence peer attachment and that the subsequent long-term unmet relationship with peer partners may result in eventual suicidal ideation.

The results also showed the significant effect of anhedonia on suicidal ideation in the pathway of both parental attachment-anhedonia-suicidal ideation and parental attachment-anhedonia-peer attachment-suicidal ideation. Consistent with previous studies, our results also showed that a poor quality of parental attachment could aggravate anhedonia (Horan et al., 2007; Blanchard et al., 2011). Studies have also shown that individuals with more severe anhedonia had low levels of positive affection toward themselves and others, had difficulty experiencing happiness with friends, and found it difficult to retain a relationship with their peers (Berry et al., 2007; Brown et al., 2007). fMRI studies also verified that, at an anatomical level, individuals with less meaningful friendships had low levels of activation in reward-related regions such as the striatum (Braams et al., 2014; Powers et al., 2018). Thus, on the basis of the interpersonal theory of suicide, the pathway of parental attachment-anhedonia-peer attachment-suicidal ideation has a significant impact on suicidal ideation. The pathway of parental attachment-anhedonia-suicidal ideation is also remarkable. Indeed, a large number of studies have verified the impact of anhedonia on suicidal ideation regardless of cognitive traits (Hawes et al., 2018) or brain mechanisms (Kennedy et al., 2006; Berridge and Kringelbach, 2008; Vang et al., 2010; Meerwijk et al., 2013). In conclusion, anhedonia can indirectly influence suicidal ideation, and future intervention to decrease suicidal ideation should have greater focus on anhedonia itself.

The current study findings have significant clinical implications. In particular, the relationships of adolescents with parents and peer partners need to be strong and positive, and anhedonia intervention is needed to effectively decrease suicidal ideation among adolescents. Furthermore, adolescent peer attachment is directly correlated with the time spent with peer partners (Thorlindsson and Bernburg, 2009). Thus, prevention for suicidal ideation can be better applied from peer attachment rather than parental attachment. Here, we considered the onset of suicidal ideation from the perspective of social interactions and the results indicated that institutes dealing with adolescents need to promote and foster positive parental and peer relationships among adolescents.

There are several limitations that should be noted. First, causality among the variables could not be guaranteed due to the cross-sectional study design. Longitudinal studies are needed to explore the relationship between parental attachment and suicidal ideation. Second, the sample size was taken from a single city, which may have decreased its generalizability. And out of respect for entrance pressure, we lost data of grades 9 and 12, which may confusion results. Third, although the model was significant, the effect sizes were relatively small. Suicidal ideation could be impacted by a variety of other variables and should be considered within an integrative theoretical framework, covering the aspects of developmental, environmental, social, psychological, psychiatric, medical, molecular, genetic, demographic, and vocational factors (Van Orden et al., 2010; Elman et al., 2013). Finally, future studies should use comprehensive measurements covering multiple factors.

In conclusion, our study shed light on the impact of attachment on suicidal ideation. The results suggested that parental attachment had a significant impact on suicidal ideation. Both anhedonia and peer attachment significantly mediated the relationship between parental attachment and suicidal ideation. The results can be applied to both educational and clinical practices. Future studies should further explore other potential underlying mechanisms to better decrease the prevalence of suicidal ideation.

## Data Availability Statement

The raw data supporting the conclusions of this article will be made available by the authors, without undue reservation.

## Ethics Statement

The studies involving human participants were reviewed and approved by HSY-IRB-PJ-HFYYYX-008, NCT03991572. Written informed consent to participate in this study was provided by the participants’ legal guardian/next of kin.

## Author Contributions

YG and YJ: design work of paper, data collection, and data analysis. YG: wrote the manuscript. YH, MJ, YL, YC, LZ, and KW: data analysis. FY and CZ: design work, data collection, and manuscript revision.

## Funding

Application of Medical Projects in Hefei Municipal Health Commission 2019 (172).

## Acknowledgments

First and foremost, I would like to show my deepest gratitude to my companion. Since everybody tried their best to complete this study. And thanks all of my friends. Last but not least, I’d like to thank all of my paper reviewer, editor et al., for their patient work on my study.

## Conflict of Interest

The authors declare that the research was conducted in the absence of any commercial or financial relationships that could be construed as a potential conflict of interest.

## Publisher’s Note

All claims expressed in this article are solely those of the authors and do not necessarily represent those of their affiliated organizations, or those of the publisher, the editors and the reviewers. Any product that may be evaluated in this article, or claim that may be made by its manufacturer, is not guaranteed or endorsed by the publisher.
